# The application of metagenomic next-generation sequencing for *Angiostrongylus* eosinophilic meningitis in a pediatric patient: A case report

**DOI:** 10.3389/fpubh.2022.1003013

**Published:** 2022-10-20

**Authors:** Jing Liu, Jinhao Tao, Weiming Chen, Tingting Wang, Xin Chen, Meili Shen, Qiuxiang Ou, Yunjian Zhang, Yifeng Ding, Jufang Wu, Xunjia Cheng, Guoping Lu, Gangfeng Yan

**Affiliations:** ^1^Pediatric Intensive Care Unit, Children's Hospital of Fudan University, National Center for Children's Health, Shanghai, China; ^2^Department of Research and Development, Nanjing Geneseeq Technology Inc., Nanjing, China; ^3^Department of Research and Development, Nanjing Dinfectome Technology Inc., Nanjing, China; ^4^Geneseeq Research Institute, Nanjing Geneseeq Technology Inc., Nanjing, China; ^5^Department of Medical, Nanjing Dinfectome Technology Inc., Nanjing, China; ^6^Department of Neurology, Children's Hospital of Fudan University, National Center for Children's Health, Shanghai, China; ^7^Institute of Antibiotics, Huashan Hospital of Fudan University, Shanghai, China; ^8^Department of Medical Microbiology and Parasitology, School of Basic Medical Sciences, Fudan University, Shanghai, China

**Keywords:** *Angiostrongylus cantonensis*, eosinophilic meningitis, metagenomic next generation sequencing, doxycycline, albendazole

## Abstract

**Background:**

*Angiostrongylus* eosinophilic meningitis (AEM) is a rare yet emerging disease caused by *Angiostrongylus cantonensis* infection. Its atypical symptoms may delay the diagnosis and cause fatal outcomes, especially in the early stages of infection and among children.

**Case presentation:**

Here we reported the use of metagenomic next-generation sequencing (mNGS) to facilitate the diagnosis and treatment of an 8-year-old boy with severe *A. cantonensis* infection. The mNGS tests consistently identified the infection of *A. cantonensis* prior to the detection by the immunologic method and confirmed it as AEM. Owing to the multidisciplinary team (MDT)-administrated treatments and close disease monitoring based on regular clinical tests and sequential mNGS tests, the patients eventually fully recovered from severe infectious conditions.

**Conclusion:**

This case demonstrated the advantages of mNGS for early diagnosis of AEM in pediatric patients, highlighting its application for pan-pathogen detection, as well as disease monitoring for severe *A. cantonensis* infection.

## Introduction

*Angiostrongylus cantonensis* (*A. cantonensis*), known as rat lungworm, transmits between its primary host of rats and intermediate hosts, including snails and slugs (1). It may infect humans upon accidental ingestion of infected intermediate hosts or contaminated raw vegetables (1, 2). *A. cantonensis* is the most common infectious cause of eosinophilic meningitis (3). *Angiostrongylus* eosinophilic meningitis (AEM) occurs mainly in the Asia-Pacific region but has spread to Europe and America due to globalism and travel-related exposure, indicating the importance of raising global awareness (4–6). The incubation period for the development of AEM is typically about 2 weeks. The presence of infiltrated eosinophils and the larvae of *A. cantonensis* in the cerebrospinal fluid (CSF) is the major pathologic characteristic (7). On the other hand, the clinical presentations of AEM vary between adults and children, whereas children have a higher incidence of nausea and vomiting, fever, lethargy, and muscle twitching and convulsions (8). The treatment of AEM is mainly supportive therapy, as the infiltrated eosinophils and other leukocytes around the worm may cause pathological inflammation and cytotrophic meningoencephalitis (7). Most symptoms usually disappear within 4 weeks of onset (9), but patients with a heavy parasite load may progress to severe symptoms, coma, and even death. Therefore, early detection and diagnosis are essential (10). At present, the diagnosis of AEM mainly depends on traditional testings, such as blood routine test (BRT), cerebrospinal fluid (CSF) cell counting, serum ELISA, qPCR, and head magnetic resonance imaging (MRI) (11). However, these techniques have limitations, such as the low throughput and lack of anti-*Angiostrongylus* immunoglobulins in the early stages, leading to unsatisfying detection rates (6). Moreover, the atypical symptoms in children may lead to misdiagnosis as viral encephalitis, particularly when the patients had no clear parasite-contact history (7). Metagenomic next-generation sequencing (mNGS) is an emerging tool that outperforms the traditional tests for hypothesis-free, pan-pathogen detection (12). Previously, the genome of *A. cantonensis* lab strains and field isolates has been reliably detected by the NGS approach (13). Here, we reported a case of an 8-year-old boy who was initially diagnosed with viral encephalitis due to inconsistent clinical manifestations and later affirmed as AEM by the detection of *A. cantonensis* in CSF using mNGS. Owing to the early pathogen detection and close disease monitoring using mNGS, the patient eventually completely recovered from severe infection symptoms.

## Case presentation

**Figure 1:**
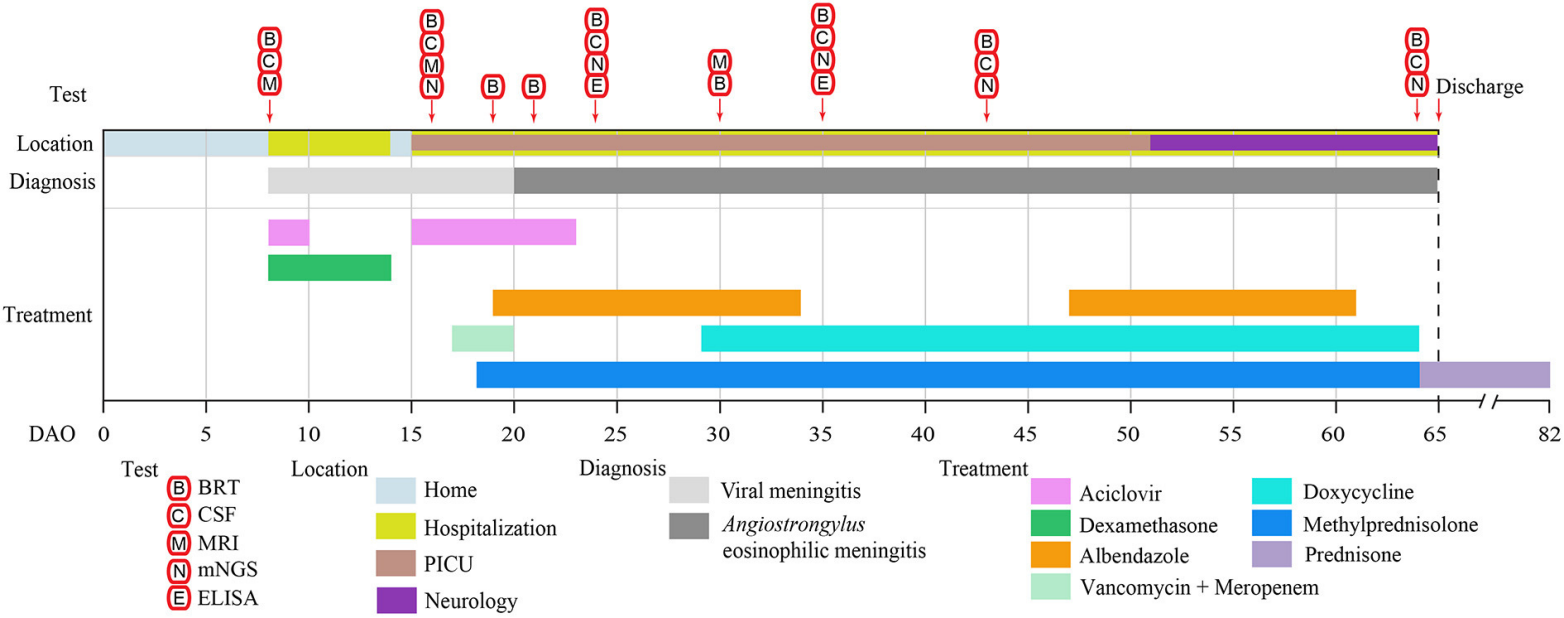
The clinical test and treatment timeline of the patient. The location, diagnosis, treatment, and duration are represented by the colored bars. The clinical tests and their operating days are indicated by the arrows. BRT, blood routine test; CSF, cerebrospinal fluid; MRI, magnetic resonance imaging; mNGS, metagenomic NGS; ELISA, enzyme-linked immunosorbent assay; DAO, day after symptom onset; PICU, Pediatric Intensive Care Unit.

An eight-year-old boy started to show low-grade fever, paroxysmal headache, mental fatigue, and loss of appetite on March 23, 2021 (Day after symptom onset, DAO 0), and was admitted to the hospital on DAO 8 due to the persistent symptoms. He had no history of hepatitis, tuberculosis, or genetic/metabolic disease. After admission, the patient received clinical examination and laboratory tests (Figure 1). Blood and CSF analysis showed that the patient had leukocytosis (white blood cell [WBC] count: 1.79 × 10^10^/L in peripheral blood, 4.63 × 10^8^/L in CSF, Table 1), indicative of central nervous system (CNS) infection. Diffuse slow wave activity on the electroencephalogram (EEG, 0.5–3.0 Hz) and abnormal MRI staining with enlargement of gyri in both frontal lobes suggested a case of virus encephalitis. Thus, acyclovir (250mg, Q8H, 2 days) and dexamethasone (0.3mg/kg, 6 days) were used. After the anti-viral treatment, the patient still experienced intermittent fever, spiritual lethargy, and aggravated headache. The autoimmune antibody testing was performed, and the negative results excluded the possibility of autoimmune encephalitis.

**Table 1:** Laboratory test results of peripheral blood and cerebrospinal fluid during hospitalization.

**Test (unit)**	**DAO 8**	**DAO 16**	**DAO 19**	**DAO 21**	**DAO 24**	**DAO 30**	**DAO 35**	**DAO 42**	**DAO 64**	**Reference range^a^**
**Peripheral blood**
WBC (× 10^9^/L)	**17.92**	**14.90**	**12.27**	**11.78**	**14.73**	**16.37**	**10.81**	9.98	**14.34**	4.00–10.00
Neutrophil (%)	**75.5**	68.8	60.9	54.4	58.2	59.5	58.5	41.2	58.1	50–70
Lymphocyte (%)	**15.1**	**9.9**	**13.9**	**16.9**	**25.6**	**28.5**	31.8	46.6	33.7	30–40
EOS (%)	N	**13.0**	**16.4**	**20.5**	**8.4**	4.8	2.2	4.0	0.5	0.5–5.0
EOS (cell/ul)	N	**1,930**	**2,010**	N	**1,240**	**790**	240	**400**	70	60–300
Platelet (× 10^9^/L)	**498**	393	342	305	380	**554**	**550**	**442**	394	100–400
Hemoglobin (g/L)	136	111	113	108	113	**107**	124	122	140	110–160
**CSF**
WBC (× 10^6^/L)	**463**	**340**	N	N	**20**	N	**100**	**70**	**78**	0–15
Monocyte (%)	80.0% (80/100)	50.0% (50/100)	N	N	50.0% (10/20)	N	70.0% (70/100)	90.0% (63/70)	89.7% (70/78)	–
Multinucleated cell (%)	20.0% (20/100)	50.0% (50/100)	N	N	50.0% (10/20)	N	30.0% (30/100)	10.0% (7/70)	10.3% (8/78)	–
Protein (mg/L)	340	**650.0**	N	N	445.7	N	**668.4**	**731.0**	358.7	<450
Glucose (mmol/L)	2.9	**1.5**	N	N	**2.0**	N	**0.4**	**1.2**	**2.3**	2.5–4.4

Abnormal results outside the reference ranges are highlighted in bold. ^a^Reference ranges according to the hospital laboratory instructions. DAO, day after symptom onset; WBC, white blood cell; EOS, eosinophils; CSF, cerebrospinal fluid; N, not tested.

**Figure 2:**
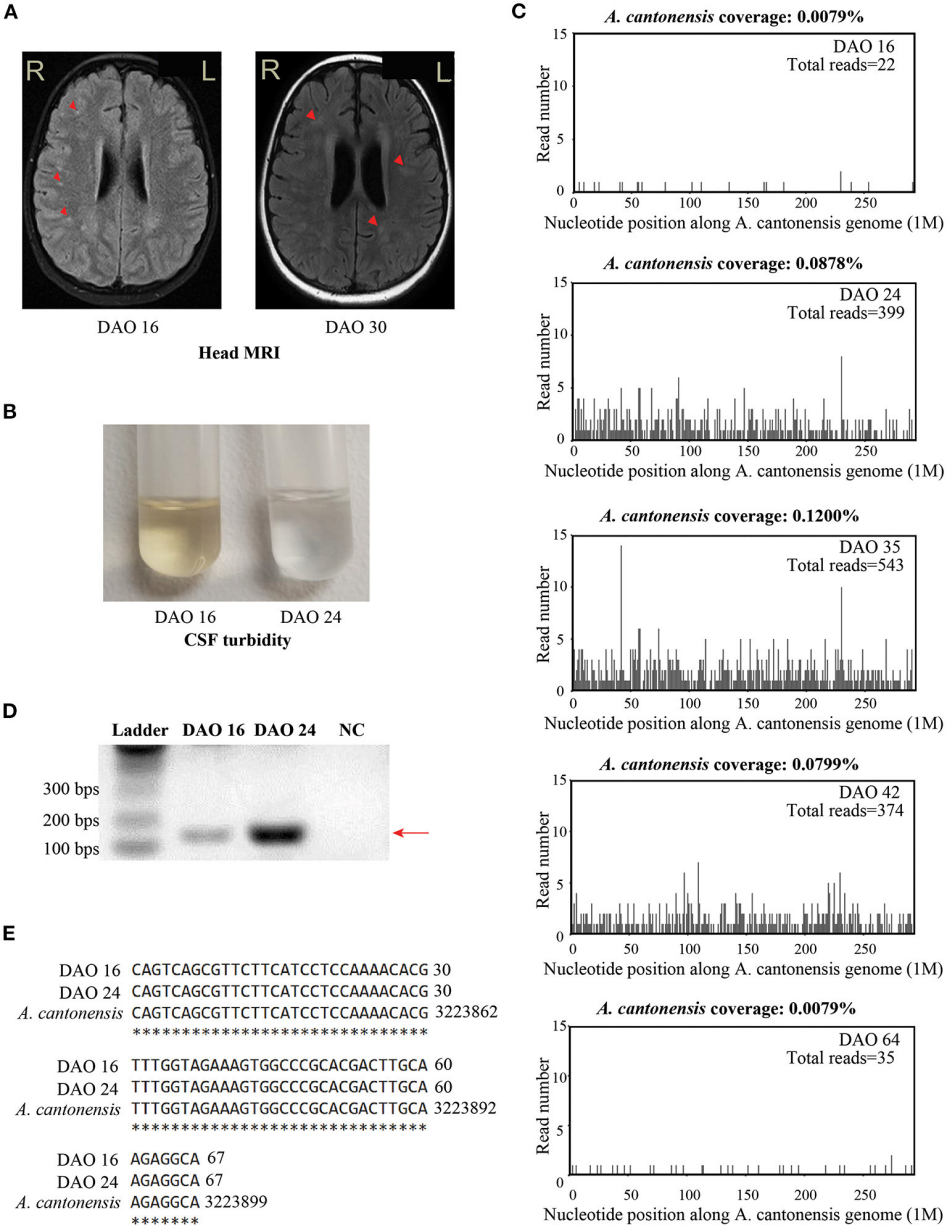
The results of clinical tests during the treatment. **(A)** The brain MRI staining on DAO 16 and DAO 30. **(B)** The turbidity of CSF samples collected on DAO 16 and DAO 24. **(C)** The patient's CSF mNGS coverages mapped to *A. cantonensis* genome on DAO 16, DAO 24, DAO 35, DAO 42, and DAO 64. **(D)** The diagram showing the bands of *A. cantonensis-*specific PCR from the CSF samples on DAO 16 and DAO 24 for Sanger sequencing. PCR primers sequences were designed against the genome sequence of *A. cantonensis*, given the sequencing result of the mNGS test: reverse primer: 5′-AAACTGTCTTGCGGACACCT-3′; forward primer: 5′-AAAGGCTCGTGTACCGCATT-3′. The size of the PCR product is 168 bps, as pointed by the arrow, which is gel purified and subject to Sanger sequencing. Ultrapure water was used as the negative control. **(E)** Sanger sequencing alignment results between the purified PCR products and *A. cantonensis* DNA sequences (NCBI: txid6313) on DAO 16 and DAO 24. The asterisks indicate the consensus sequences. DAO, day after symptom onset; NC, negative control.

On DAO 15, the patient's condition worsened with disturbance of consciousness and no response to pain stimulus. The Glasgow Coma Scale (GCS) score assessment was 6. He was immediately admitted to Pediatric Intensive Care Unit (PICU), Children's Hospital of Fudan University. Acyclovir (250mg, Q8H, 8 days), vancomycin (0.65g, Q8H, 3 days), and meropenem (1.3g, Q8H, 3 days) were used (Figure 1). Given the deteriorating condition after anti-viral treatment, we further investigated the contact history of the patient and a pathogen hosts exposure (touching/handling snails in the backyard) was reported, suggesting pathogenic infection. Therefore, further laboratory tests of CSF were conducted on DAO 16 for pathogen identification. BRT results showed WBC at 1.49 × 10^10^/L (68.8% neutrophils, 9.9% lymphocytes, and 13.0% eosinophils) and eosinophils up to 1,930 cells/ul, which was much higher than the clinical reference range (Table 1). The CSF analysis showed that WBC was 3.4 × 10^8^/L (50% monocytes and 50% multinucleated cells), and CSF protein and glucose were 650 and 1.5 mg/L, respectively (Table 1). Head MRI found bilateral temporal lobe hyperintensity (BTH) and small round patchy hyperintensity in lateral ventricles (Figure 2A). Microscopic observation found CSF turbidity (Figure 2B) but the CSF culture test failed to identify any pathogens. In addition, the DNA detection of enterovirus and herpes simplex virus 1 and 2 (HSV-1 and 2) were all negative. Thus, we performed an mNGS test with CSF sample in a Clinical Laboratory Improvement Amendments-certified and College of American Pathologists-accredited laboratory (Nanjing Geneseeq Technology, Jiangsu Province, China) as described previously (14), which revealed *A. cantonensis* DNA sequences covering 0.0079% of the genome (Figure 2C). Sanger sequencing confirmed the presence of *A. cantonensis* DNA sequences (Figures 2D,E). No other potential pathogenic microbe sequence was detected. It is worth mentioning that the mNGS detection of *A. cantonensis* infection preceded the CSF ELISA tests, whereas the latter yielded inconsistent results (DAO 24: negative; DAO 35: weakly positive), as also noted by others. Collectively, the patient was diagnosed with AEM. The standard treatment of albendazole (0.2 g, bid, 15 days) and methylprednisolone (40 mg, qd, 23 days; 20 mg, qd, 8 days; 40 mg, qd, 17 days) were administered (Figure 1).

The patient's body temperature was reduced upon the standard AEM treatment, but he remained unconscious. Another round of laboratory tests and CSF mNGS was performed on DAO 24. Peripheral blood eosinophils and CSF WBC (Table 1) exhibited a rapid reduction, and the CSF became clear (Figure 2B). However, peripheral blood WBC was still high, and the *A. cantonensis* genome coverage (0.0878%) increased according to CSF mNGS (Figure 2C). As the patient was still in severe condition, we held a multidisciplinary team (MDT) meeting with experts from the departments of infectious diseases and pediatric neurology, as well as the institute of antibiotics. Due to its use in treating filarial infections, doxycycline (0.064 g, bid) was added from DAO 29. The BRT on DAO 30 showed symptom relief with decreased eosinophils and increased lymphocytes (GCS score = 12). On DAO 30, MRI scanning detected several abnormal signals in the right cerebellar hemisphere and the bilateral occipital lobe (Figure 2A). Due to the side effects of albendazole, only doxycycline and methylprednisolone were administered after DAO 34. Regular laboratory tests and mNGS were used to monitor the patient (Figure 1). On DAO 42, peripheral blood eosinophils slightly increased, the proportion of CSF WBC (10% monocytes and 90% multinucleated cells) appeared abnormal (Table 1), and CSF mNGS still detected abundant genomic DNA sequences of *A. cantonensis* (Figure 2C). Therefore, albendazole (0.2 g, bid) was added back. The symptoms were gradually resolved. On DAO 51, the patient was discharged from PICU and transferred to the Department of Neurology to continue the treatment. When discharged on DAO 65, the patient's body temperature had been normal for over 2 weeks. He was free of nervous system symptoms. The GCS assessment score was 14, all indicators returned to the reference range except the mildly high peripheral blood and CSF WBC levels (Table 1), and the *A. cantonensis* DNA coverage dropped drastically by CSF mNGS (Figure 2C). After discharge, the patient was on prednisone (30 mg, qd, 5 days; 25 mg, qd, 3 days; 20 mg, qd, 3 days; 15 mg, qd, 3 days; 10 mg, qd, 3 days; 5 mg, qd, 3 days). One-year follow-up indicated that the patient has fully recovered from AEM with no sign of neurological consequences.

## Discussion

Here we have shown mNGS as a powerful molecular diagnostic tool for AEM. The sensitive detection of *A. cantonensis* DNA sequences in patients' CSF facilitated clinical practice. Traditional etiological detection methods of parasitic infection are usually time-consuming and have low sensitivity. For human infection with *A. cantonensis* larva, the first case in mainland China was reported in 1984 (15). AEM is difficult to diagnose and treat and could become fatal, with long-term sequelae of blindness and limb paralysis and a fatality rate of up to 10% in children (16). Early diagnosis and intervention are crucial, as untreated or delayed treatment may cause severe CNS angiostrongyliasis with mortality as high as 79-91% (17). The rate of detecting *A. cantonensis* larvae from the CSF samples of patients with angiostrongyliasis is still low (18). The diagnosis of AEM based on clinical manifestations, microscopic and ELISA detection, and MRI scanning is often challenging (19). For instance, ELISA has been commonly used for identifying *A. cantonensis* in patients with angiostrongyliasis, but the accuracy is not optimal for early-stage patients and children (7, 20). Due to its atypical symptom, AEM in children could be easily misdiagnosed as viral encephalitis, particularly in patients with undetermined exposures to the parasites. Besides, eosinophil abnormality in peripheral blood and CSF may also be sophisticated and caused by other infectious diseases and cancers (21, 22). Real-time PCR is another powerful approach to detect microbial pathogens with the ability of quantitation. A previous study has also demonstrated the detection of *A. cantonensis* DNA in human CSF specimens by real-time PCR from 22 out of 33 eosinophilic meningitis patients (11). However, for patients with acute illnesses of unknown etiology, mNGS has become a new precise diagnostic technique for pan-pathogen detection (23). Appropriate mNGS tests showed clinical significance in detecting parasitic infections *via* CSF (24). A previous case reported the positive *A. cantonensis* detection in CSF using mNGS after initial negative results of regular blood and CSF tests, which was 11 days prior to the ELISA confirmation (17). In our case, the patient was initially diagnosed with viral CNS infection due to atypical symptoms. Based on his clinical manifestations, we suspected it was a rare pathogenic infection and conducted mNGS of CSF. Specific DNA reads corresponding to *A. cantonensis* were detected by mNGS as early as DAO 16, while ELISA testing was negative on DAO 24 and weakly positive on DAO 35. We then combined the evidence from mNGS, laboratory testing, clinical manifestations, and the patient's contact history to reach a confident diagnosis and quick response, representing an effective approach to implementing mNGS in clinical practice. Furthermore, the coverages of *A. cantonensis-*specific mNGS reads helped us determine the infection condition and guide appropriate treatments. The *A. cantonensis* read coverage increased alongside disease development, remained high during severe symptoms, and started to decrease during the relief of neurological symptoms. When discharged, low coverage of *A. cantonensis* reads was still detected, which may reflect the process of pathogenic DNA removal. It is also worth noting that due to the technical and regulatory obstacles, mNGS remains in the early stage of clinical adoption without a consensus on detection threshold standards. Both the experimental procedures and the computational analysis pipeline largely affect the specificity and sensitivity of pathogen detection by mNGS (25, 26). The detection of pathogens with very low concentrations was extremely difficult and several procedure optimizations, such as microbe nucleic acid enrichment and host nucleic acid depletion, were developed (27). Our findings shed light on pan-pathogen profiling for neurological infections using CSF mNGS, which could promote the application of mNGS under urgent situation.

For the treatment of AEM, about 80% of the cases received anthelmintics, and 20% received therapeutic lumbar punctures (LPs) (5). As safe and broad-spectrum anthelmintics, albendazole and benzimidazole are often preferred for treating *A. cantonensis* infection (28). However, dead worms in the brain could cause severe inflammatory reactions and induce headaches or brain damage (29). Dexamethasone has been added to the treatment but is controversial due to its adverse effects (30). As a broad-spectrum bacteriostatic agent, doxycycline is an FDA-approved tetracycline antibiotic targeting the 30S ribosomal subunit (31). It has been used as an anti-inflammatory drug for pathogenic infection by reducing inflammatory cytokines such as IL-1b, IL-6, and TNF-α (32). Due to its anti-inflammatory properties, doxycycline has been used with albendazole to treat lymphatic filariasis infection (33, 34). Indeed, albendazole combined with doxycycline has shown additive benefits against onchocerciasis (35). However, doxycycline is not recommended by FDA for use in children under 8 years of age except for severe conditions with the potential side effects of tooth discoloration and enamel hypoplasia. In the presented case, the addition of doxycycline suggested by the MDT was due to the unimproved situation but whether doxycycline significantly contributed to the AEM recovery remained to be investigated beyond this single case. Notably, the pediatric patient showed good tolerance to doxycycline combined with standard albendazole plus methylprednisolone treatment.

## Conclusion

In conclusion, our case has demonstrated the use of mNGS for the early detection of *A. cantonensis* infection in a pediatric patient of undetermined etiology by traditional methods. The early diagnosis using mNGS has facilitated the appropriate and timely treatments for improved prognosis and more studies are warranted to evaluate the ability of mNGS in early detection and disease monitoring in larger cohorts as well as in the clinical practice.

## Data availability statement

The datasets for this article are not publicly available due to concerns regarding participant/patient anonymity. Requests to access the datasets should be directed to the corresponding authors.

## Ethics statement

Ethical review and approval was not required for the study on human participants in accordance with the local legislation and institutional requirements. Written informed consent to participate in this study was provided by the participants' legal guardian/next of kin.

## Author contributions

JL, JT, GL, and GY designed the study. JL, JW, and XCheng attended the patient. JL, JT, and MS analyzed the clinical data. JT, WC, YZ, and YD collected samples and performed the tests. XChen, TW, and QO wrote the manuscript. GL and GY revised the manuscript and supervised the study. All authors have read and approved the final manuscript.

## Funding

This study was funded by the National Key Research and Development Program of China (2021YFC2701800 and 2021YFC2701805).

## Conflict of interest

Authors TW, XChen, and QO are employees of Nanjing Geneseeq Technology Inc., China. Authors TW and MS are employees of Nanjing Dinfectome Technology Inc., China. The remaining authors declare that the research was conducted in the absence of any commercial or financial relationships that could be construed as a potential conflict of interest.

## Publisher's note

All claims expressed in this article are solely those of the authors and do not necessarily represent those of their affiliated organizations, or those of the publisher, the editors and the reviewers. Any product that may be evaluated in this article, or claim that may be made by its manufacturer, is not guaranteed or endorsed by the publisher.
